# Supplementary material for: Histone methyltransferase ASH1L primes metastases and metabolic reprogramming of macrophages in the bone niche
Source: Nat Commun. 2025 May 20;16:4681. doi: 10.1038/s41467-025-59381-2 (PMC12092585; doi:10.1038/s41467-025-59381-2)
Supplement: Supplementary file 1 — Supplementary Information [file 41467_2025_59381_MOESM1_ESM.pdf]

Supplementary Information

Histone Methyltransferase ASH1L Primes Metastases and Metabolic Reprogramming of Macrophages in the Bone Niche

Chenling Meng[1], Kevin Lin[2], Wei Shi[1], Hongqi Teng[1], Xinhai Wan[3], Anna DeBruine[1,4], Yin Wang[1], Xin Liang[1], Javier Leo[1,4], Feiyu Chen[1], Qianlin Gu[1], Jie Zhang[1], Vivien Van[5], Kiersten L. Maldonado[5], Boyi Gan[1], Li Ma[1], Yue Lu[2*], Di Zhao[1*]

This PDF file includes

- Supplementary Figures 1-10

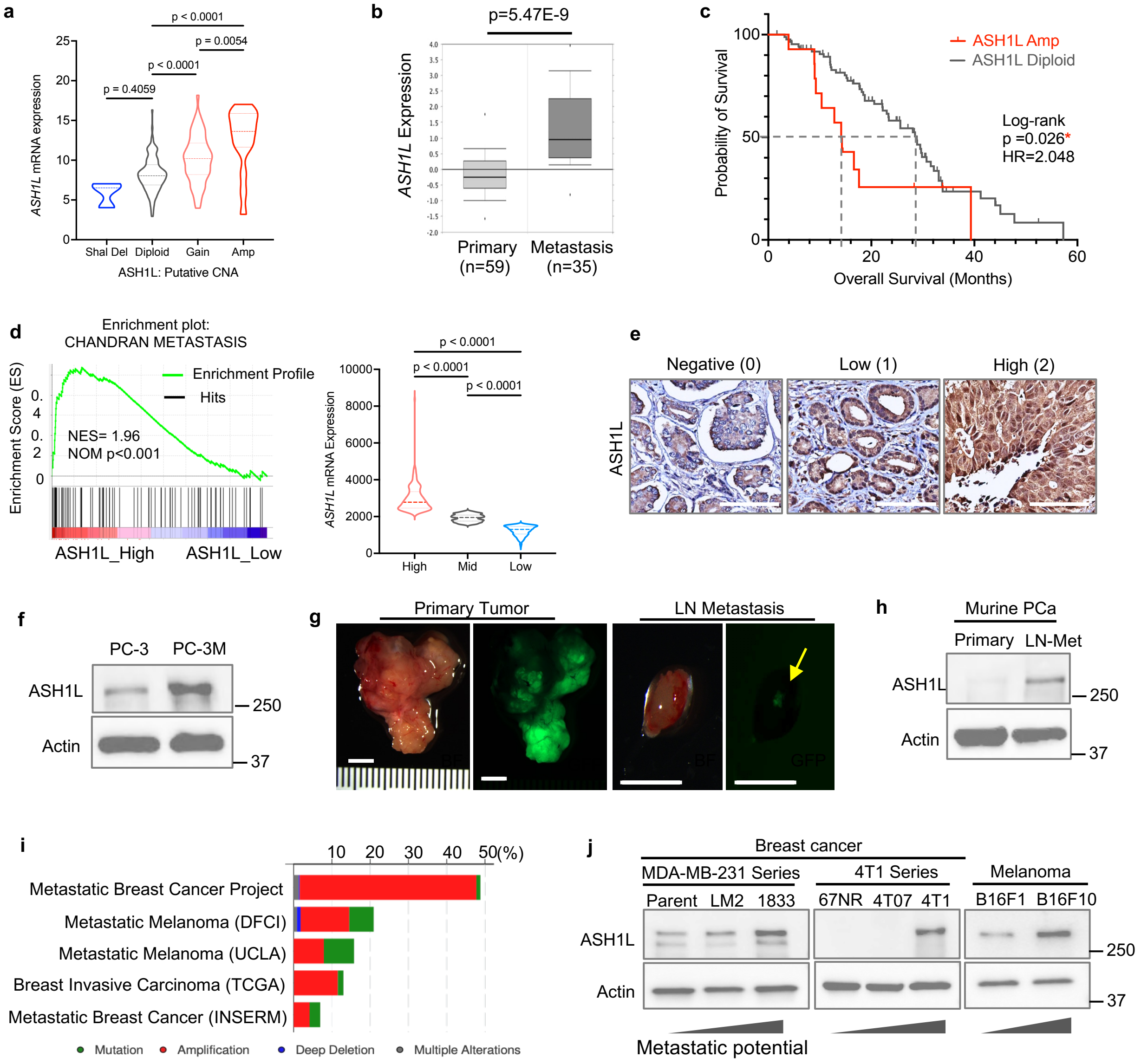

Supplementary Fig. 1 ASH1L is genetically amplified and overexpressed in metastatic cancers

a, *ASH1L* mRNA expression in metastatic tumors with genetic alterations of *ASH1L*. **b,** Log2 Median-centered Ratio of ASH1L expression in primary versus metastatic prostate tumors (Grasso dataset). The Oncomine™ Platform (Thermo Fisher, Ann Arbor, MI) was used for analysis and visualization. n=59 for primary tumors. n=35 for metastatic tumors. **c,** Overall survival of metastatic PCa patients (SU2C), with or without ASH1L amplification. The log-rank (Mantel-Cox) test was used for statistical analysis. **d,** Based on ASH1L mRNA expression, 493 PCa samples in the TCGA dataset were classified into ASH1L_high (n=150), ASH1L_mid (n=193), and ASH1L_low (n=150) groups. The enrichment of metastasis profile in ASH1L_high versus ASH1L_low samples was determined by GSEA. The normalized Enrichment Score (NES) and Nominal p-value (NOM p) are shown. **e,** Representative images of ASH1L IHC staining in human PCa samples. Scale bar = 100μm. **f,** Western blot analysis of ASH1L in human PCa cell lines with different metastatic potential. ASH1L and Actin were run in the same gel. **g,** Representative GFP and bright-field (BF) images of primary prostate tumor and lymph node (LN) metastasis from *Pb-Cre; Pten$^{L/L}$; Trp53$^{L/L}$; Smad4$^{L/L}$; mTmG (PbPPS)* mice. Scale bar = 4 mm. **h,** Western blot analysis of ASH1L in primary and LN metastatic tumors derived from *PbPPS* mice. The samples derive from the same experiment but different gels for ASH1L, another for Actin were processed in parallel. **i,** Frequencies of *ASH1L* genetic alterations in human metastatic malignancies (cbioportal). **j,** Western blot analysis of ASH1L in breast and melanoma cancer cells with different metastatic potential. ASH1L and Actin were run in the same gel within different series. Statistical significance was determined by unpaired two-tailed T-test (**b**) or One-way ANOVA with Tukey's post hoc test (**a** and **d**). Data in **b** represent the mean ± standard deviation. The experiments in **f**, **h**, and **j** were repeated independently three times, yielding similar results. Source data are provided as a Source Data file.

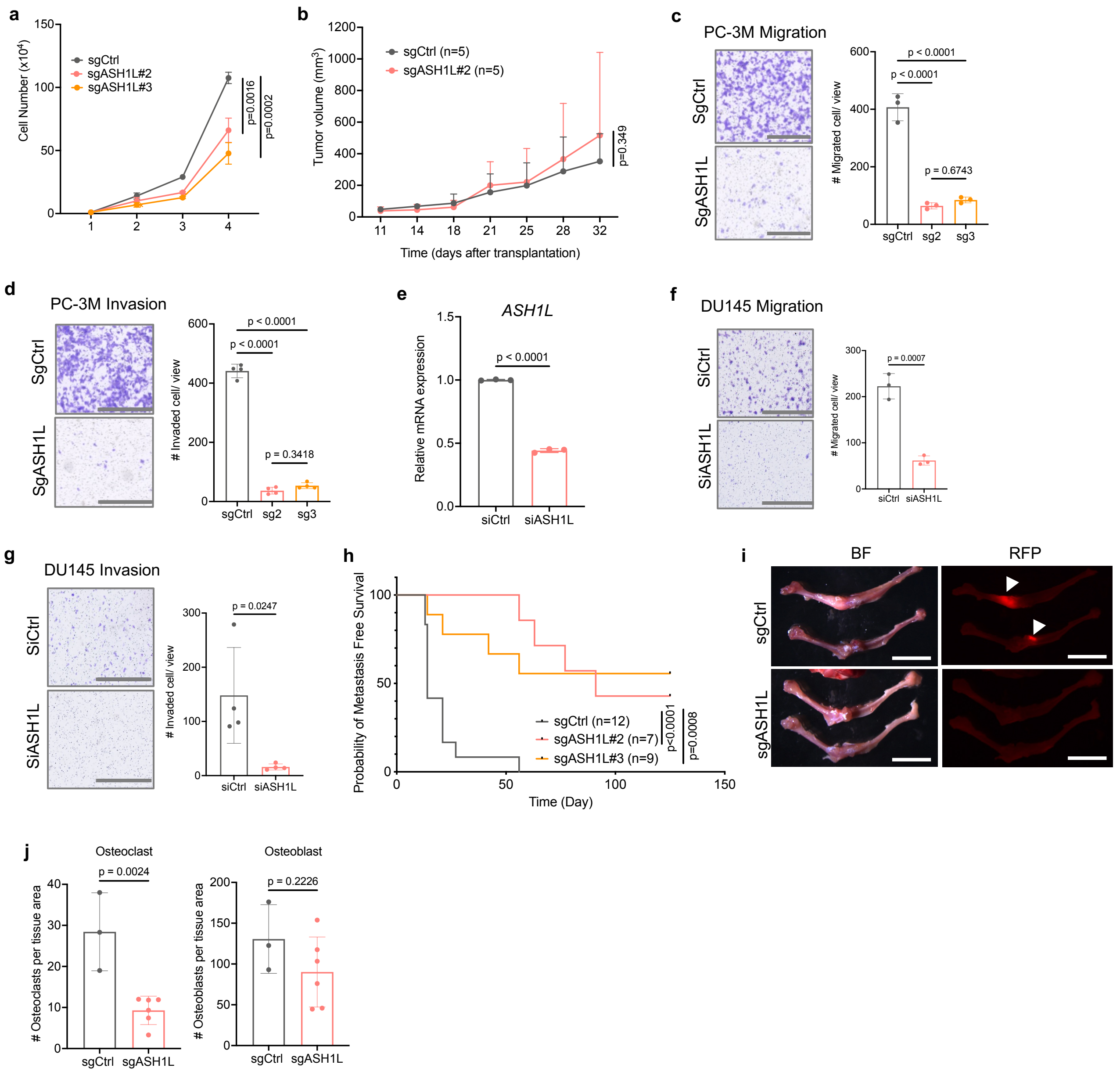

Supplementary Fig. 2 Depletion of ASH1L suppresses cell invasiveness and bone metastasis.

a,b, Proliferation (**a**) and tumor growth (**b**) of control and ASH1L-depleted PC-3M cells. n = 3 biological replicates per group in **a.** n=5 mice per group in **b**. **c,d,** Representative images and quantification of migrated (**c**) and invaded (**d**) control and ASH1L-depleted PC-3M cells. Quantification is based on n=3 biological replicates per group in **c,** n=4 biological replicates per group in **d**. **e,** Expression of ASH1L in DU145 cells transfected with siRNAs for 72 h, determined by qPCR. n = 3 biological replicates per group. **f,g,** Representative images and quantification of migrated (**f**) and invaded (**g**) control and ASH1L-depleted DU145 cells. Quantification is based on n=3 biological replicates per group in **f,** n=4 biological replicates per group in **g**. **h,** Kaplan-Meier metastasis-free survival of mice. The Log-rank (Mantel-Cox) test was used for statistical analysis. n=12, 7 and 9 mice in each group, as indicated. **i,** Representative bright field (BF) and Ex vivo fluorescence of bone tissues from mice transplanted with control and ASH1L-depletion PC-3M cells. Scale bar =1cm. **j,** Quantification analysis of osteoclasts (left) or osteoblasts (right) per mm^2 bone tissues of mice intracardiac injected with control and ASH1L-depleted PC-3M cells. n=3 mice (sgCtrl), n=6 mice (sgASH1L). Statistical significance was determined by unpaired two-tailed T-test (**b, e, f, g,** and **j**) or One-way ANOVA with Tukey's post hoc test (**a, c,** and **d**). Data in **a-g** and **j** represent the mean ± standard deviation. Source data are provided as a Source Data file.

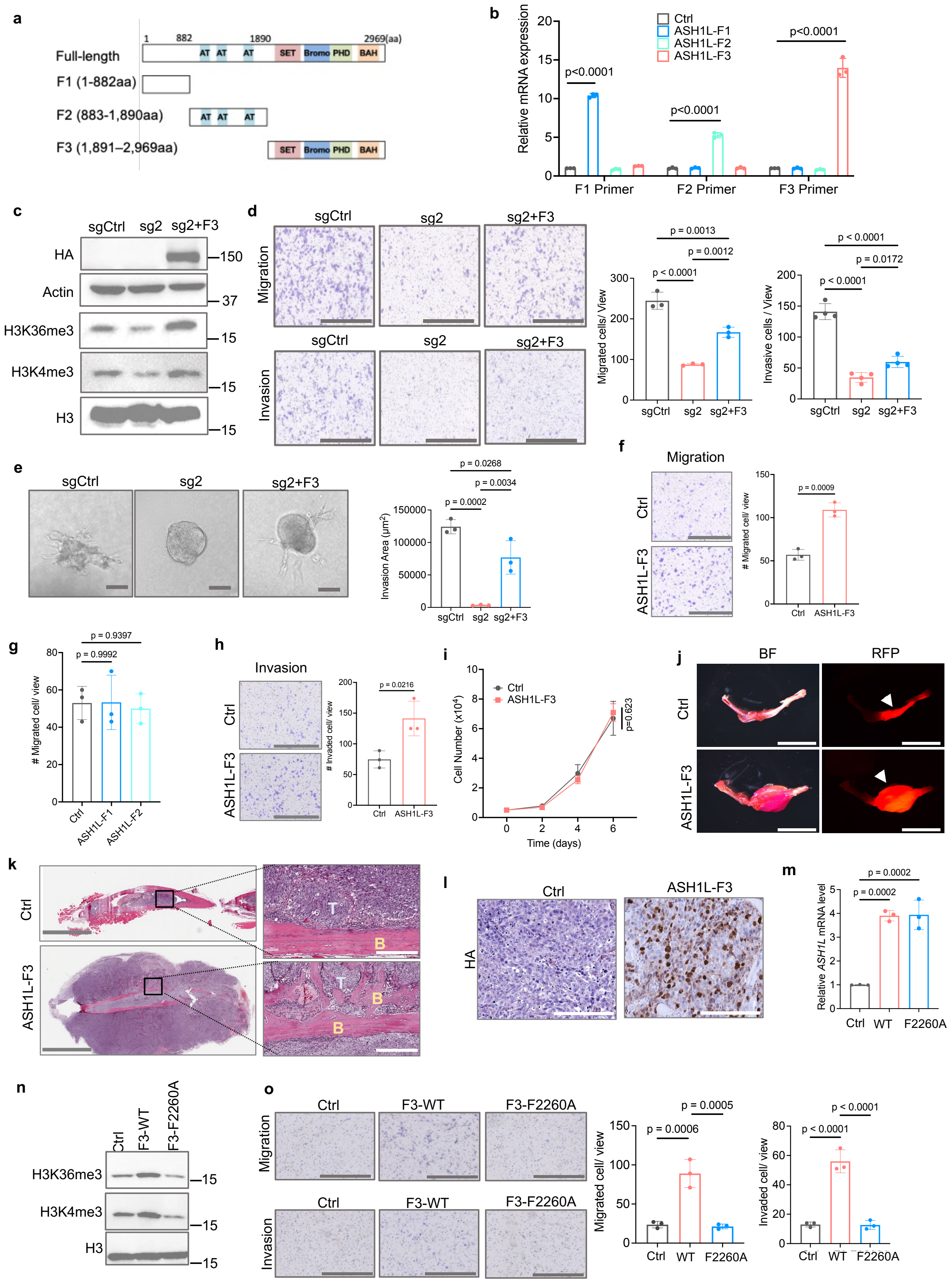
a
Full-length
1
882
1890
2969(aa)
AT AT AT SET Bromo PHD BAH
F1 (1-882aa)
F2 (883-1,890aa)
F3 (1,891–2,969aa)
b
Relative mRNA expression
Ctrl
ASH1L-F1
ASH1L-F2
ASH1L-F3
p<0.0001
F1 Primer
F2 Primer
F3 Primer
c
sgCtrl sg2 sg2+F3
HA
Actin
H3K36me3
H3K4me3
H3
150
37
15
d
sgCtrl sg2 sg2+F3
Migration
Invasion
p = 0.0013
p = 0.0012
p < 0.0001
Migrated cells/ View
p < 0.0001
p = 0.0172
Invasive cells / View
e
sgCtrl sg2 sg2+F3
p = 0.0268
p = 0.0034
p = 0.0002
Invasion Area (μm²)
f
Migration
Ctrl
ASH1L-F3
p = 0.0009
# Migrated cell/ view
g
p = 0.9397
p = 0.9992
# Migrated cell/ view
Ctrl ASH1L-F1 ASH1L-F2
h
Invasion
Ctrl
ASH1L-F3
p = 0.0216
# Invaded cell/ view
i
Ctrl
ASH1L-F3
p=0.623
Cell Number (x10⁴)
Time (days)
j
BF RFP
Ctrl
ASH1L-F3
k
Ctrl
ASH1L-F3
T
B
l
Ctrl ASH1L-F3
HA
m
p = 0.0002
p = 0.0002
Relative ASH1L mRNA level
Ctrl WT F2260A
n
Ctrl F3-WT F3-F2260A
H3K36me3
H3K4me3
H3
15
o
Ctrl F3-WT F3-F2260A
Migration
Invasion
p = 0.0005
p = 0.0006
Migrated cell/ view
p < 0.0001
p < 0.0001
Invaded cell/ view
Ctrl WT F2260A

Supplementary Fig. 3 ASH1L overexpression promotes PCa invasion and bone metastasis.

a, Schematics of full-length human ASH1L and truncated proteins (F1, F2, and F3) containing different domains. **b,** Expression of truncated ASH1L determined by qPCR. n=3 biological replicates per group. **c,** ASH1L-F3 was reintroduced in ASH1L-depleted PC-3M cells, followed by western blot analyses of HA and H3 marks. The samples were derived from the same experiment, but different gels for HA and Actin, another for H3, another for H3K4me3, and another for H3K36me3 were processed in parallel. **d,** Representative images and quantification of migrated and invaded control, ASH1L-depleted, and ASH1L-F3 reintroduced PC-3M cells. Scale bar = 1000 μm. n=3 biological replicates per group for migration assay, n=4 biological replicates per group for invasion assay. **e,** 3D sphere invasion assays of control, ASH1L-depleted and ASH1L-F3 reintroduced PC-3M cells. Scale bar = 100μm. Quantification is based on n=3 biological replicates per group. **f,** Representative images and quantification of migrated LNCaP cells overexpressing ASH1L-F3. Scale bar = 1000 μm. n=3 biological replicates per group. **g,** Quantification of migrated LNCaP cells overexpressing ASH1L-F1 or ASH1L-F2. Quantification is based on n=3 biological replicates per group. **h,** Representative images and quantification of invaded LNCaP cells overexpressing ASH1L-F3. Scale bar = 1000 μm. Quantification is based on n=3 biological replicates per group. **i,** Proliferation of control and ASH1L-F3 overexpressing LNCaP cells *in vitro*. n=3 biological replicates per group **j,** Representative BF and RFP images of tumor-bearing tibias in control and ASH1L-F3 groups. Scale bar = 1cm. **k,** H&E staining of tumor-bearing tibias at different magnifications. Scale bar = 4 mm (left, low magnification) or 300 μm (right, high magnification). T: tumor; B: bone. **l,** IHC staining of HA in tibial tumors expressing vector control and HA-tagged ASH1L-F3. Scale bar = 200μm. **m,** Expression of ASH1L determined by qPCR in LNCaP cells expressing vector control, wild-type or F2260A mutated ASH1L-F3. n=3 biological replicates per group. **n,** Western blot analyses of H3 marks LNCaP cells expressing vector control, wild-type, or F2260A mutated ASH1L-F3. The samples derived from the same experiment but different gels for H3, another for H3K4me3, and another for H3K36me3 were processed in parallel. **o,** Representative images and quantification of migrated and invaded cells expressing vector control, wild-type or F2260A mutated ASH1L-F3. Scale bar = 1000 μm. n=3 biological replicates per group. Statistical significance was determined by unpaired two-tailed T-test (**f, h,** and **i**) or One-way ANOVA with Tukey's post hoc test (**b, d, e, g, m,** and **o**). Data in **b, d-i, m,** and **o** represent the mean ± standard deviation. The experiments in **c** and **n** were repeated independently three times, yielding similar results. Source data are provided as a Source Data file.

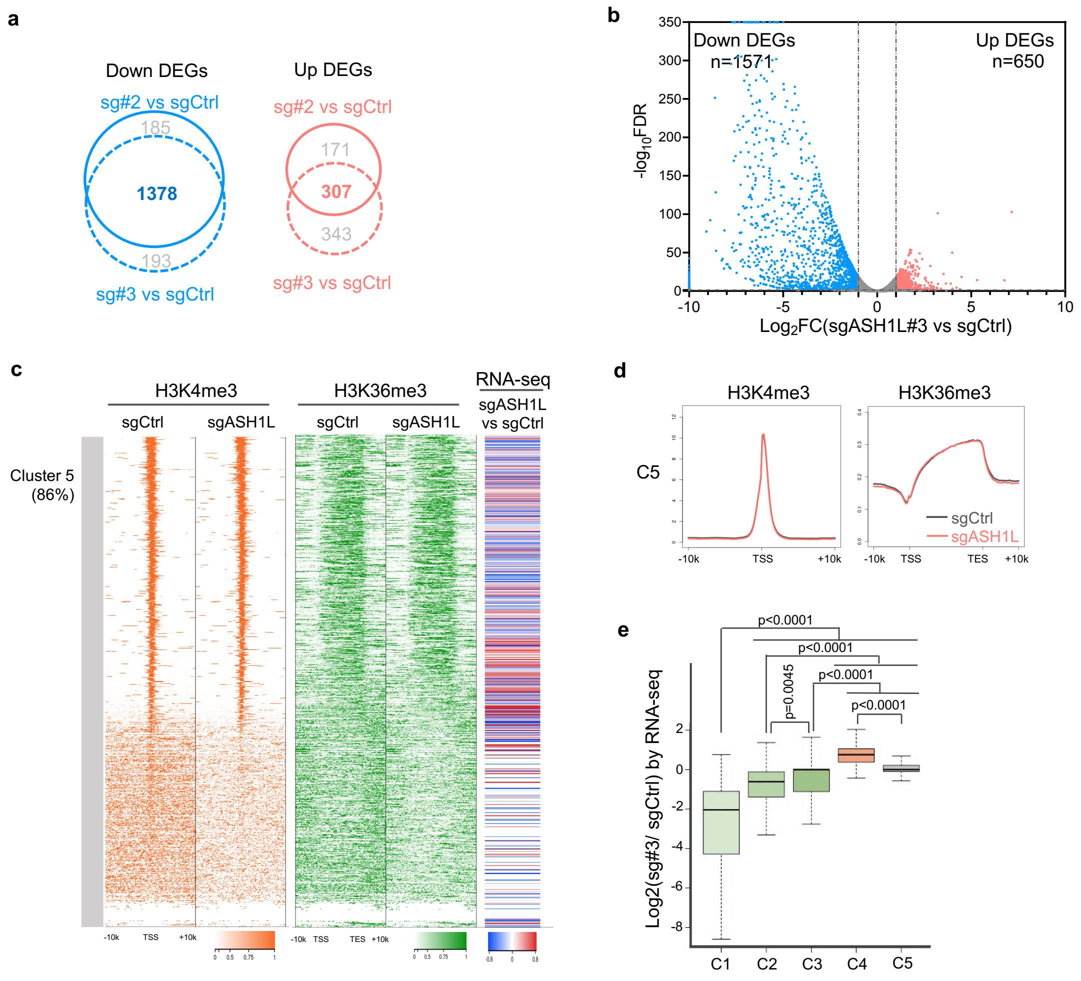

Supplementary Fig. 4 ASH1L reprograms pro-metastatic transcriptome via modulating methylations at H3K4 and H3K36.

a, Venn diagram illustrating the overlap of protein-coding DEGs in sg#2 and sg#3 PC-3M cells. 1378 down-regulated DEGs and 307 up-regulated DEGs (FDR ≤ 0.05 and FC ≥ 2) were identified from both sgRNAs targeting ASH1L. **b,** Volcano plot illustrating the results of differential expression analysis comparing control (sgCtrl) versus ASH1L-depleted (sg#3) PC-3M cells. Each dot represents a protein-coding gene, with up-regulated (n=1571) and down-regulated (n=650) genes shown in red and blue, respectively. Horizontal and vertical dashed lines indicate thresholds of FDR = 0.05 and FC = 2, respectively. **c,** Heatmaps illustrating H3K4me3 (orange) and H3K36me3 (green) marks over TSS or body of genes in control and ASH1L-depleted PC-3M cells, determined by CUT&RUN. Genes were grouped into 5 clusters (C1-C5) by changes in H3K4me3 and H3K36me3 signals. Heatmaps of C5 (no changes in either H3K4me3 or H3K36me3) are shown. The right column color-coded in red (upregulated) or blue (downregulated) represents the log2 fold change of gene expression in sgASH1L#2 versus sgCtrl cells (RNA-seq). **d,** Metaplots illustrating the average signal distribution of H3K4me3 (left) and H3K36me3 (right) over genes in C5. The signals in control (gray) and ASH1L-depleted (red) PC-3M cells are compared. Transcription start sites (TSS) and transcript end sites (TES) are indicated. **e,** Box plots illustrating the log2FC of gene expression in sgASH1L#3 versus sgCtrl cells for the five clusters. Each box represents the interquartile range (IQR; 25th to 75th percentile), the center line indicates the median, and whiskers extend to the most extreme data points within 1.5×IQR. Statistical comparisons between clusters were performed using two-sided unpaired Welch's t-tests. n=580 genes (C1); n=1042 genes (C2); n=750 genes (C3); n=384 genes; n=16875 genes (C5). Data in **e** represent the mean ± standard deviation. Source data are provided as a Source Data file.

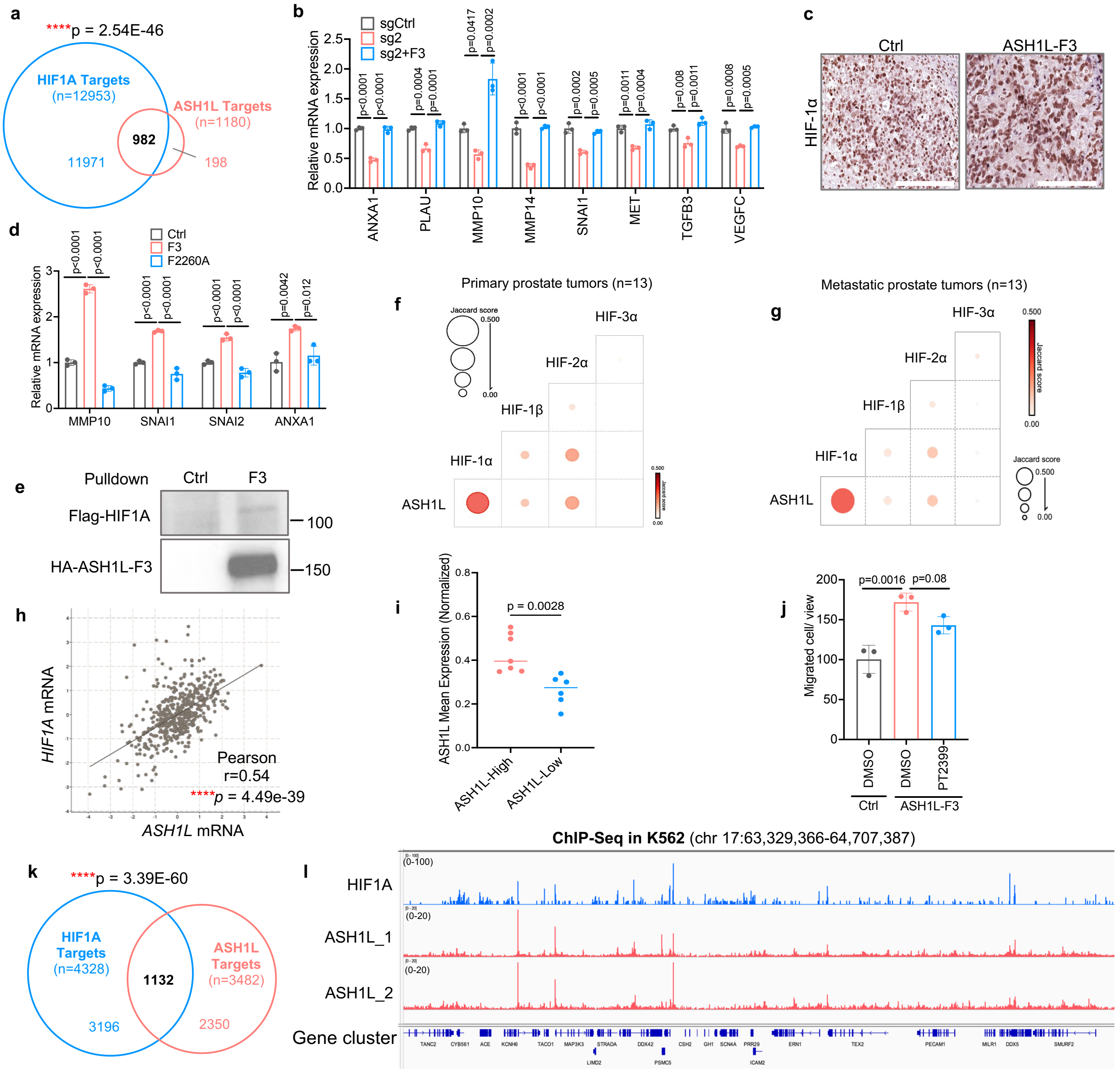

Supplementary Fig. 5 ASH1L co-opts with HIF-1α induce pro-metastatic genes and enhance invasiveness

a, Venn diagram displaying co-target genes of HIF1A and ASH1L in metastatic PCa cells. The blue circle presents HIF1A-bound genes in PC-3 cells, determined by ChIP-seq (GSE106305; n=12953); the red circle presents ASH1L target genes identified in PC-3M cells as shown in Fig. 2f (n=1180). **b,** Expression of ASH1L target genes in control, ASH1L-depleted, and ASH1L-F3 reintroduced PC-3M cells. n=3 biological replicates per group. **c,** IHC staining of HIF-1α in bone tumors derived from control and ASH1L-F3 overexpressing LNCaP. Scale bar = 200μm. **d,** Expression of ASH1L targets in LNCaP expressing vector control, wild-type or F2260A mutated ASH1L-F3. n=3 biological replicates per group. **e,** HA-tagged ASH1L-F3 protein and Flag-tagged HIF1A protein were overexpressed and purified, followed by pull-down assays using HA-beads. The interaction between two proteins was determined by Western blot assay. The samples were derived from the same experiment, but different gels for Flag-HIF1A and another for HA-ASH1L-F3 were processed in parallel. **f,g,** Correlation analysis of ASH1L and HIF family gene expression in epithelial cells from primary tumors (**f,** *Chen et al. 2021,* GSE141445) and metastatic prostate tumors (**g,** *Chan et al. 2022,* GSE210358*)*, determined by scRNA-seq. **h,** Scatter plot illustrating the correlation between ASH1L and HIF1A expression in human PCa tumor samples (TCGA). The Pearson correlation coefficient (r) and *P* value are reported. **i,** 13 patients with primary PCa (GSE141445) were classified into two groups (ASH1L_Low vs ASH1L_High) by normalized ASH1L mean expression levels in epithelial cells. **j,** Control and ASH1L-F3-overexpressing LNCaP cells were treated with DMSO or HIF-2α inhibitor PT2399 (10 μM) for 48h, followed by migration assays. n = 3 biological replicates. **k,** Venn diagram displaying co-target genes of HIF1A and ASH1L in leukemia cell line K562. The blue and red circles present genes whose promoters were bound with HIF1A (GSE123461; n= 4328) or ASH1L (ENCSR115BBC; n= 3482), respectively, determined by ChIP-seq assays. **l,** Representative ChIP-seq tracks illustrating the enrichments of HIF1A (blue; GSE123461) and ASH1L (red; ENCSR115BBC) at the indicated gene cluster (chr 17:63,329,366-64,707,387) in K562 leukemia cells. Fisher's exact test (**a** and **k**), unpaired two-tailed Student's t-test (**i**), and one-way ANOVA with Tukey's post hoc test (**b, d** and **j**) were used for statistical analysis. Data in **b, d** and **j** represent the mean ± standard deviation.

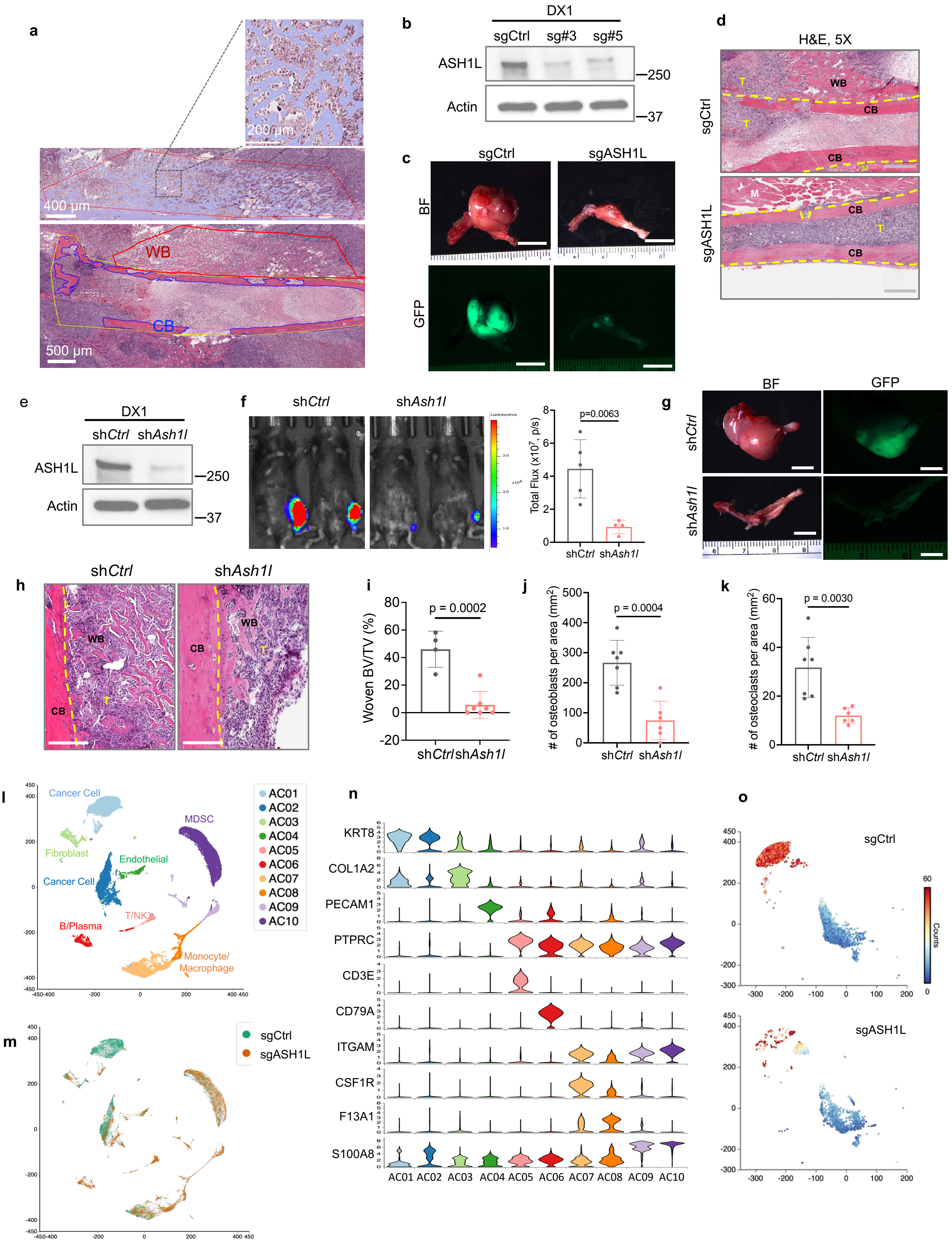
a
200 µm
400 µm
WB
CB
500 µm
b
DX1
sgCtrl sg#3 sg#5
ASH1L
Actin
—250
—37
c
sgCtrl sgASH1L
BF
GFP
d
H&E, 5X
sgCtrl
sgASH1L
WB
CB
T
M
e
DX1
shCtrl shAsh1l
ASH1L
Actin
—250
—37
f
shCtrl shAsh1l
p=0.0063
Total Flux (x10^7, p/s)
g
BF GFP
shCtrl
shAsh1l
h
shCtrl shAsh1l
WB
CB
T
i
p = 0.0002
Woven BV/TV (%)
j
p = 0.0004
# of osteoblasts per area (mm^2)
k
p = 0.0030
# of osteoclasts per area (mm^2)
l
Cancer Cell
Fibroblast
Endothelial
MDSC
T/NK
B/Plasma
Monocyte/ Macrophage
AC01 AC02 AC03 AC04 AC05 AC06 AC07 AC08 AC09 AC10
m
sgCtrl
sgASH1L
n
KRT8
COL1A2
PECAM1
PTPRC
CD3E
CD79A
ITGAM
CSF1R
F13A1
S100A8
o
sgCtrl
sgASH1L
Counts

Supplementary Fig. 6 Depletion of ASH1L suppresses PCa metastatic tumor outgrowth in the bone in the syngeneic model.

a, Representative images showing the histopathology of tumor outgrowth in the bone model with mixed osteolytic/osteoblastic features. It also presents the area for quantifying cortical bone (CB) volume (Bottom, circled in blue) vs tissue volume (bottom, circled in yellow) and new woven bone (WB) volume (top, lavender shadow) vs tissue volume (top, circled in red). **b,** Western blot analysis of ASH1L in control and ASH1L knockout DX1 cells using the Cas9-CRISPR system. ASH1L and Actin were run in the same gel. **c,** Representative BF and GFP images of tumor-bearing tibias in control and ASH1L-knockout groups. Scale bar = 1 cm. **d,** Representative images of H&E of control and ASH1L-depleted bone tumors. Scale bar = 400 μm. T: tumor; CB: cortical bone; WB: new woven bone; M: Muscle. **e,** ASH1L was knocked down in DX1 cells using shRNA, followed by Western blot analysis. ASH1L and Actin were run in the same gel. **f,** Control and ASH1L-knockdown DX1 cells expressing firefly luciferase were injected into the tibias of C57BL/6J mice. Bioluminescence images and quantification at 3 weeks after injection are shown. (n=5 mice for control; n=4 mice for ASH1L-knockdown group)**. g,** Representative BF and GFP images of tumor-bearing tibias in control and ASH1L-knockdown groups. Scale bar = 1cm. **h,** H&E staining of control and ASH1L-knockdown tumor-bearing mice. Scale bar = 300μm. **i**, Quantification of new woven bone from control (n=4 tumors) and ASH1L-knockdown (n=7 tumors). Woven BV/TV (%): percentage of new woven bone volume in bone formation tissue area. **j**,**k**, Quantification of the number of osteoclasts (**j**) and osteoblasts (**k**) per mm^2 in the new woven bone area from control (n=7) and ASH1L-depleted (n=6) bone tumors. **l-n,** ScRNA-seq was performed in control (n=3) and ASH1L-depleted (n=4) bone tumors. The UMAP views of 30,850 single cells color-coded by ten major clusters (AC1-AC10, **l**) or two groups (**m**) are presented. The violin plots present marker gene expression for each cell cluster (**n**). **o,** tSNE views of epithelial cells from control and ASH1L-depleted bone tumors, color-coded by the count of HIF-1α target genes. Unpaired two-tailed Student's t-test was used for statistical analysis in **f** and **i-k**. Data in **f** and **i-k** represent the mean ± standard deviation. The experiments in **b**, and **e** were repeated independently three times, yielding similar results. Source data are provided as a Source Data file.

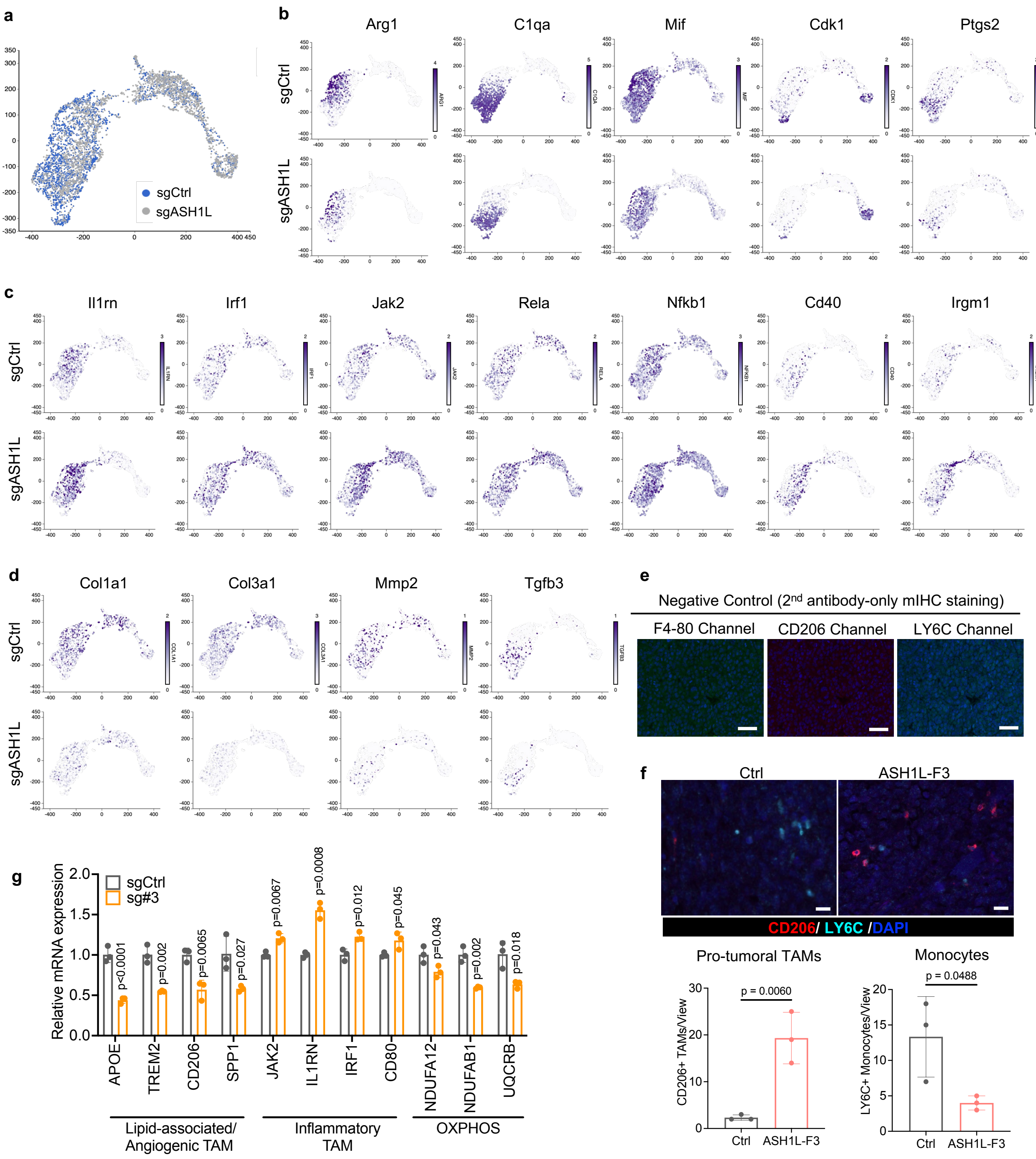

Supplementary Fig. 7 Single-cell transcriptome profiling reveals ASH1L induces macrophage plasticity in metastatic bone niche

a, The UMAP view of 3,046 Monocyte/TAM color-coded by two groups is presented with the nomination of each subcluster. **b-d,** UMAP views of TAMs from control versus ASH1L-depleted bone tumors, color-coded by expression of indicated genes. **e,** Representative images of secondary antibody-only negative control for F4-80, CD206, and LY6C mIHC staining from shCtrl bone tumors. Scale bar = 50 µm. **f,** Representative images (enlarged) of multiplex IHC staining and quantifications of pro-tumoral macrophages (CD206^{+}) and monocytes (LY6C^{+}) in bone tumors derived from control and ASH1L-overexpressing (ASH1L-F3) LNCaP cells. n=3 mice per group. Scale bar = 20µm. **g,** THP-1 cells were pretreated with PMA to induce macrophage differentiation and then co-cultured with control and ASH1L-depleted PC-3M cells for 48h using a trans-well system. mRNA expression of TAM markers and OXPHOS-related genes was determined by qPCR. n=3 biological replicates per group. Unpaired two-tailed Student's t-test was used for statistical analysis (**g** and **f**). Data represent the mean ± standard deviation. Source data are provided as a Source Data file.

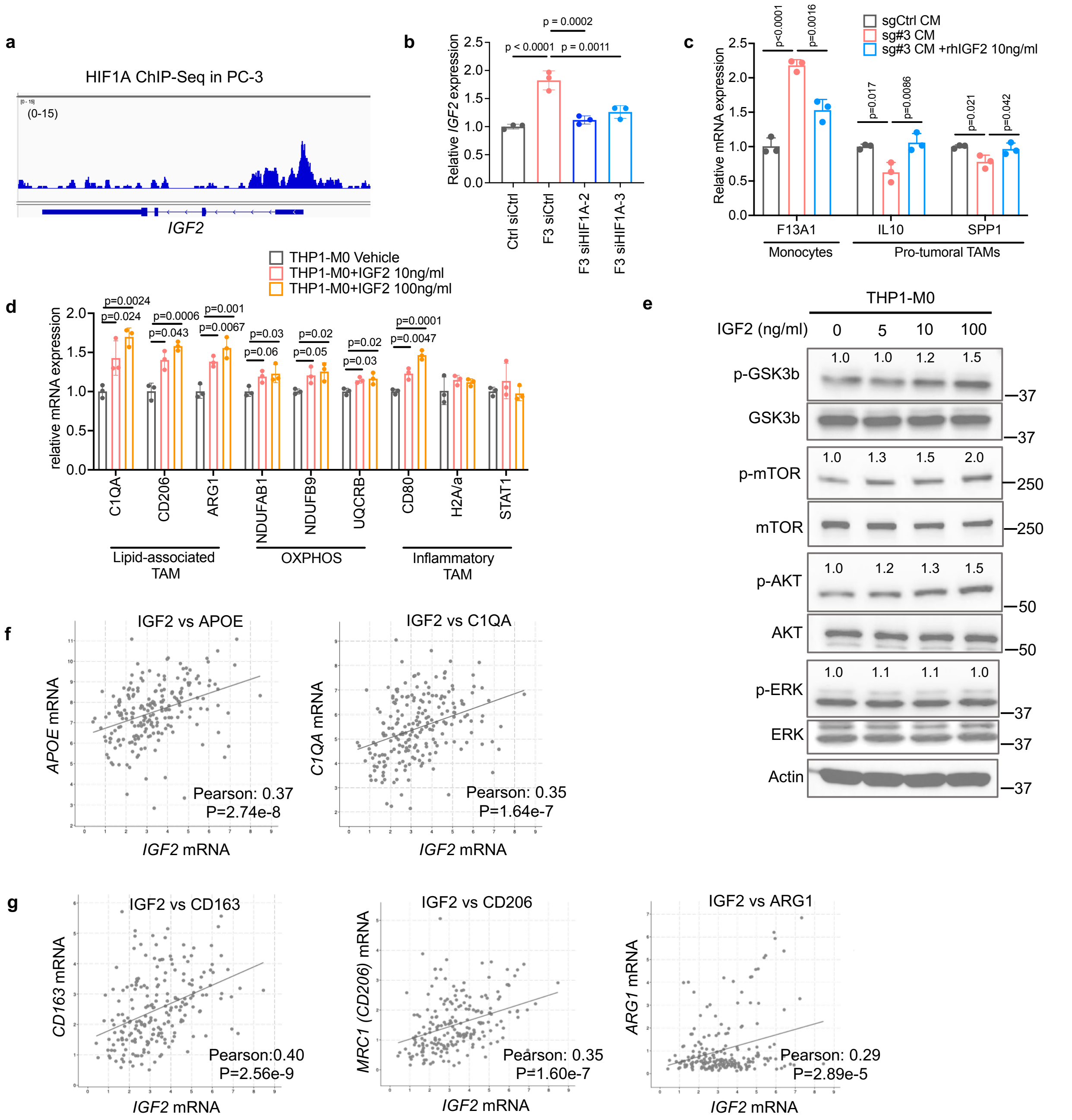

Supplementary Fig. 8 ASH1L induces lipid-associated TAMs by promoting IGF2-mediated oxidative phosphorylation

a, ChIP-seq tracks illustrating the enrichments of HIF1A at the promoter of IGF2 in PC-3 cells (GSE106305). **b,** Control or ASH1L-F3-overexpressing LNCaP cells were transfected with siRNA targeting HIF1A in the presence of CoCl2 (200uM). mRNA expression of the indicated genes was determined by qPCR. n=3 biological replicates per group. **c,** Expression of indicated genes in THP-1 cells co-cultured with conditional medium from control and ASH1L-depleted PC-3M cells with or without recombinant protein of human IGF2 (10ng/ml). n=3 biological replicates per group. **d,e,** THP-1 cells were pretreated with PMA to induce macrophage differentiation and then treated with different concentrations of IGF2 for 48h. mRNA expression of TAM markers and OXPHOS-related genes was determined by qPCR (**d**), and indicated signaling markers were determined by western blot (**e**). The ratios of phosphorylation/total protein intensities are quantified and normalized to the control (IGF2=0 ng/ml) sample. The samples were derived from the same experiment, but different gels for p-AKT, another for AKT, another for p-ERK, another for ERK, another for GSK3b and mTOR, another for p-GSK3b and p-mTOR, another for Actin were processed in parallel. n=3 biological replicates per group in **d**. **f,g,** Scatter plot illustrating the correlation of IGF2 expression with lipid-associated TAM markers (**f**) and pro-tumoral phenotype markers (**g**) in human metastatic PCa samples. The Pearson correlation coefficient (r) and P value are reported. Data was derived from the SU2C/PCF PCa dataset (n=208). one-way ANOVA with Tukey's post hoc test (**b-d**) was used for statistical analysis. Data represent the mean ± standard deviation. The experiments in **e** were repeated independently three times, yielding similar results. Source data are provided as a Source Data file.

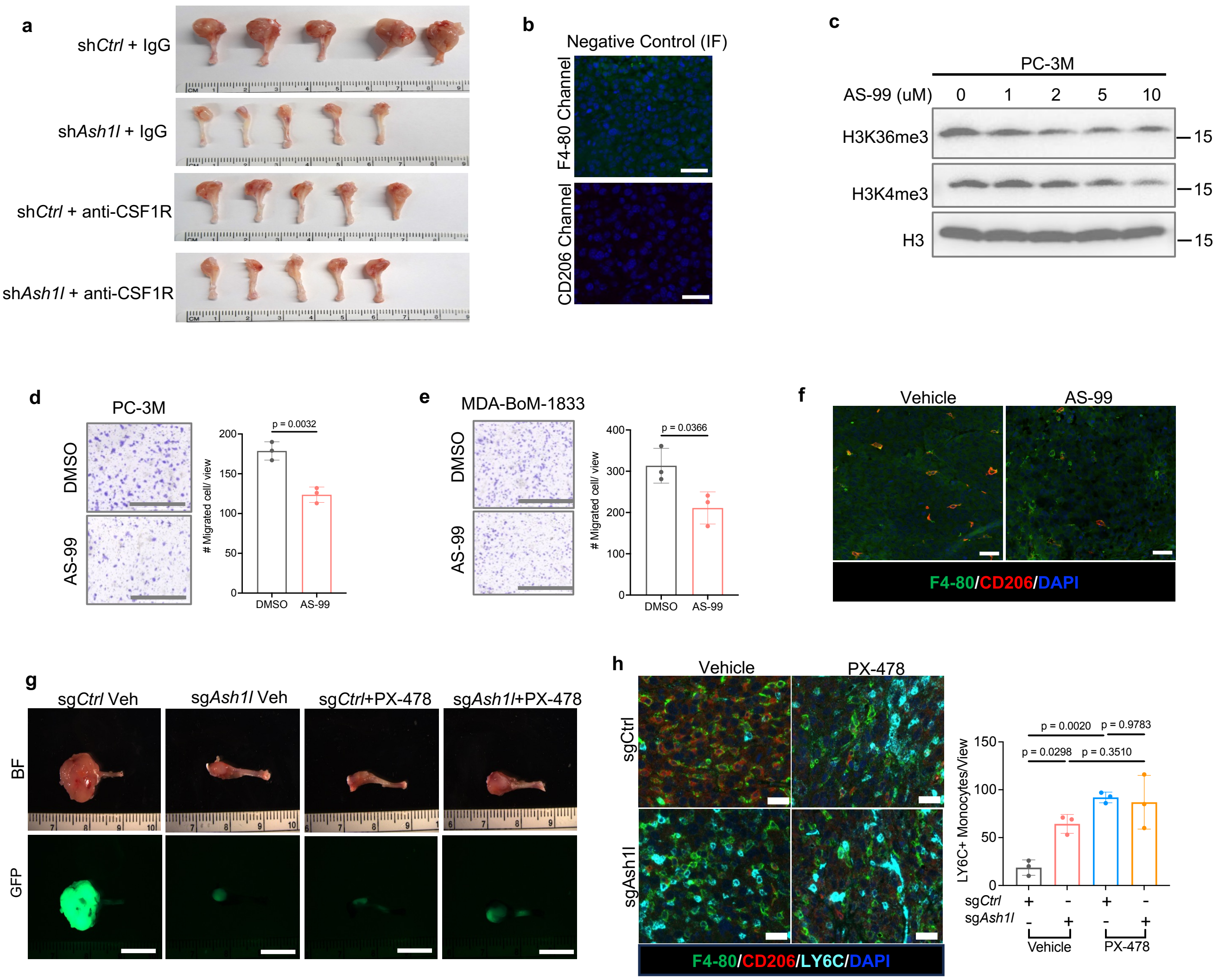

Supplementary Fig. 9 Inhibition of ASH1L-HIF-1α-TAM axis suppresses bone metastases of PCa

a, Images of tibias bearing control and ASH1L-knockdown bone tumors after treatment of IgG and anti-CSF1R antibodies. n=5 mice per group. **b,** Representative images of secondary antibody-only negative control for IF staining from shCtrl/IgG bone tumors. Scale bar = 50μm. **c,** Western blot analysis of indicated histone marks in PC-3M cells treated with ASH1L inhibitor AS-99 at different concentrations. The samples were derived from the same experiment, but different gels for H3, another for H3K4me3, and another for H3K36me3 were processed in parallel. **d,e,** Representative images and quantification of migrated PC-3M (**d**) and MDA-BoM-1833 (**e**) cells treated with DMSO or ASH1L inhibitor AS-99 (10μM) for 24 h. n=3 biological replicates per group. Scale bar = 1000 μm. **f,** Representative images of TAMs in metastatic tumors upon AS-99 treatment, determined by Multiplex IHC staining. Scale bar = 50 μm. **g,** Representative BF and GFP images of control and ASH1L-knockout bone tumors after treatment of vehicle (Veh) or PX-478. Scale bar =1cm. **h,** Representative images (enlarged) of pro-tumoral TAMs (F4-80+CD206+) and pro-inflammatory TAMs (F4-80+CD206-), and monocytes (LY6C+) in control and ASH1L-depleted bone tumors with or without ASH1L inhibitor PX-478 treatment, determined by multiplex IHC staining. Scale bar = 50μm. Quantification of monocytes is shown (right). n=3 mice per group. Unpaired two-tailed Student's t-test (**d** and **e**) and one-way ANOVA with Tukey's post hoc test (**h**) were used for statistical analysis. Data in **d-e**, and **h** represent the mean ± standard deviation. The experiments in **c** were repeated independently three times, yielding similar results. Source data are provided as a Source Data file.

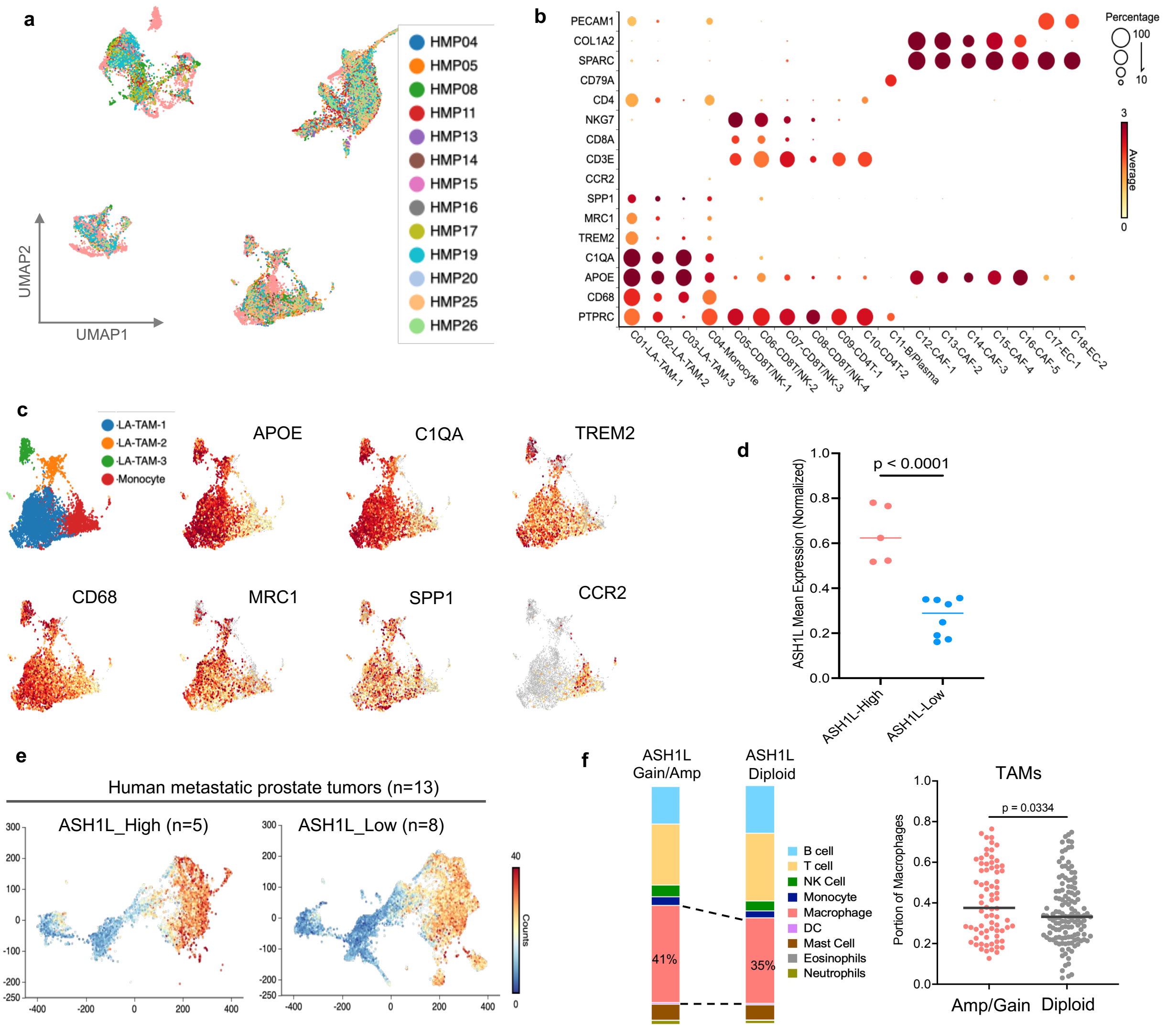

Supplementary Fig. 10 Clinical relevance of ASH1L expression with HIF1A transcriptome and TAMs in metastatic PCa.

a-e, Single-cell transcriptomic analysis of human PCa metastatic tumors (GSE210358; n = 13 patients). **a,** The UMAP view of 24,433 non-epithelial cells color-coded by 13 patients. **b,** The bubble plot presents marker gene expression for each subcluster, where dot size and color represent the percentage of marker gene expression (Percentage) and the averaged scaled expression (Average) value, respectively. LA-TAM, lipid-associated TAM; CAF, cancer-associated fibroblast; EC, endothelial cells. **c,** UMAP views of myeloid cells, color-coded by assigned cell types or indicated gene expression. **d,** 13 patients were classified into two groups (ASH1L_Low vs ASH1L_High) by normalized ASH1L mean expression levels in epithelial cells (Low n=8; High n=5). **e,** tSNE views of epithelial cells color-coded by the count of HIF-1α target genes. **f,** Proportions of immune cells, including TAMs, in metastatic tumors with gain/amplification of *ASH1L* gene, determined by CIBERSORT. Data was derived from the SU2C/PCF metastatic PCa dataset. Unpaired two-tailed Student's t-test (**d** and **f**) was used for statistical analysis. Source data are provided as a Source Data file.
