# Supplementary material for: Histone methyltransferase ASH1L primes metastases and metabolic reprogramming of macrophages in the bone niche
Source: Nat Commun. 2025 May 20;16:4681. doi: 10.1038/s41467-025-59381-2 (PMC12092585; doi:10.1038/s41467-025-59381-2)
Supplement: Supplementary file 2 — Description of Additional Supplementary Files [file 41467_2025_59381_MOESM2_ESM.pdf]

Description of Additional Supplementary Files

Supplementary Data 1. DEGs upon ASH1L KO FDR0.05 FC2.xlsx

RNA-sequencing was performed in sgASH1L (#2 and #3) vs sgCtrl PC-3M cells. n=3 biological replicates per group. Differentially expressed genes were identified in sgASH1L vs sgCtrl PC-3M (FDR<0.05 and FC>2) using unpaired two-tailed Student's t-test.

Supplementary Data 2. CUT&RUN of H3K4me3 FDR0.05 FC1.5.xlsx

H3K4me3 CUT&RUN-sequencing was performed in sgASH1L vs sgCtrl PC-3M cells. n=2 biological replicates per group. Differentially enriched peaks were identified in sgASH1L vs sgCtrl PC-3M (FDR<0.05 and FC>1.5) using edgeR, which applies a two-sided test based on the negative binomial distribution.

Supplementary Data 3. CUT&RUN of H3K36me3 FDR0.05 FC1.5.xlsx

H3K36me3 CUT&RUN-sequencing was performed in sgASH1L vs sgCtrl PC-3M cells. n=2 biological replicates per group. Differentially enriched peaks were identified in sgASH1L vs sgCtrl PC-3M (FDR<0.05 and FC>1.5) using edgeR, which applies a two-sided test based on the negative binomial distribution.

Supplementary Data 4. ASH1L direct target genes in PC-3M cells (n=1180).xlsx

The downregulated DEGs (FDR ≤ 0.05; FC ≥ 1.5) with decreased H3K4me3 and/or H3K36me3 signals (FDR ≤ 0.05; FC ≥ 1.5) in ASH1L-depleted vs control PC-3M cells (*Some metastasis-associated genes are highlighted)

Supplementary Data 5. HIF-1a ASH1L co-targets in metastatic PCa cells and K562 cells.xlsx

Tab 1: 982 overlapping genes of ASH1L targets we identified in PC-3M cells (n=1180; supplementary table 4) and HIF-1a targets determined by ChIP-seq in PC-3 cells (n=11971; GSE106305) (*Some metastasis-associated genes are highlighted);

Tab 2: 1132 overlapping genes of ASH1L targets (n=3482; ENCSR115BBC) and HIF-1a targets (n=4328; GSE123461) determined by ChIP-seq in K562 cells

Tab 3: 37 overlapping genes of ASH1-HIF-1a co-targets in leukemia (n=1132) and metastatic PCa (n=982)

Supplementary Data 6. Immune Components Marker Identification.xlsx

ScRNA-seq was performed in control (n=3 mice) and ASH1L-depleted (n=4 mice) bone tumors. PTPRC+ immune cells were sub-clustered and analyzed. 17,423 immune cells were clustered into nine subclusters (C1-C9). The differential expression analyses of cells in each cluster versus remaining cells were performed using two-tailed T-tests (reasoning that some clusters contain much fewer cells than others)

Supplementary Data 7. Mono-TAM Subcluster Marker Identification.xlsx

ScRNA-seq was performed in control (n=3 mice) and ASH1L-depleted (n=4 mice) bone tumors. 3,046 Monocytes and TAMs were clustered into seven subclusters (MC1-MC7). The differential expression analyses of cells in each cluster versus remaining cells were performed using two-tailed T-tests (reasoning that some clusters contain much fewer cells than others)

Supplementary Data 8. DEG Mono-TAM sgASH1l-sgCtrl-Wilcoxon.xlsx

ScRNA-seq was performed in control (n=3 mice) and ASH1L-depleted (n=4 mice) bone tumors. Differentially expressed genes of TAMs in ASH1L-depleted versus control tumors (FDR < 0.05; FC > 1.5) were identified using a two-tailed Wilcoxon test.

Supplementary Data 9. siRNA sgRNA shRNA plasmids and primers.xlsx

List of oligonucleotides (siRNA, sgRNA, shRNA, primers) and plasmids used in this studies

Supplementary Data 10. Gene Sets.xlsx

Genesets used for GSEA analysis and colored UMAP in this study
