# Supplementary material for: Histone methyltransferase ASH1L primes metastases and metabolic reprogramming of macrophages in the bone niche
Source: Nat Commun. 2025 May 20;16:4681. doi: 10.1038/s41467-025-59381-2 (PMC12092585; doi:10.1038/s41467-025-59381-2)
Supplement: Supplementary file 13 — Reporting Summary [file 41467_2025_59381_MOESM13_ESM.pdf]

nature portfolio

Corresponding author(s): Di Zhao

Last updated by author(s): Apr 14, 2025

Reporting Summary

Nature Portfolio wishes to improve the reproducibility of the work that we publish. This form provides structure for consistency and transparency in reporting. For further information on Nature Portfolio policies, see our Editorial Policies and the Editorial Policy Checklist.

Statistics

For all statistical analyses, confirm that the following items are present in the figure legend, table legend, main text, or Methods section.

n/a	Confirmed	
☐	☒	The exact sample size (n) for each experimental group/condition, given as a discrete number and unit of measurement
☐	☒	A statement on whether measurements were taken from distinct samples or whether the same sample was measured repeatedly
☐	☒	The statistical test(s) used AND whether they are one- or two-sided<br>*Only common tests should be described solely by name; describe more complex techniques in the Methods section.*
☒	☐	A description of all covariates tested
☐	☒	A description of any assumptions or corrections, such as tests of normality and adjustment for multiple comparisons
☐	☒	A full description of the statistical parameters including central tendency (e.g. means) or other basic estimates (e.g. regression coefficient) AND variation (e.g. standard deviation) or associated estimates of uncertainty (e.g. confidence intervals)
☐	☒	For null hypothesis testing, the test statistic (e.g. F, t, r) with confidence intervals, effect sizes, degrees of freedom and P value noted<br>*Give P values as exact values whenever suitable.*
☒	☐	For Bayesian analysis, information on the choice of priors and Markov chain Monte Carlo settings
☒	☐	For hierarchical and complex designs, identification of the appropriate level for tests and full reporting of outcomes
☐	☒	Estimates of effect sizes (e.g. Cohen's d, Pearson's r), indicating how they were calculated

Our web collection on statistics for biologists contains articles on many of the points above.

Software and code

Policy information about availability of computer code

Data collection	Aperio CS2 (Leica Biosystems) , dissection microscopy((Leica Biosystems), fluorescent cell imager (Bio-Rad) were used to acquire images.<br>ChemiDoc Imaging System (Bio-Rad) was used for Western blot analysis.<br>QuantStudio 3 Real-Time PCR Systems (Thermo Fisher Scientific) was used for qPCR.<br>IVIS 200 and IVIS Lumina XR (PerkinElmer) was used for detecting Bioluminescence signals.<br>MX-20 Cabinet X-ray System was used for monitoring bone structure.<br>Cytation 5 was used for whole insert scanning of 2D migration and invasion assays.
Data analysis	ImageJ was used to analyze 3D cell invasion area/length and count cells for 2D migration and invasion assays.<br>For clinical relevance data, TCGA data from Bioportal was use. The correlations of high ASH1L expression with Gleason scores (Tomlins dataset) and metastasis (Grasso dataset) in PCa were analyzed and visualized using the OncomineTM Platform (Thermo Fisher, Ann Arbor, MI).ASH1L putative copy number alterations and mRNA expression (FPKM capture) data on 444 metastatic PCa tumors (SU2C) (3) and 489 primary PCa tumors (TCGA) were downloaded from cbioportal (www.cbioportal.org). The putative copy number alterations in theASH1L gene include shallow deletion, diploid, gain, and amplification. The copy number alterations of ASH1L in primary (TCGA) versus metastatic (SU2C) prostate tumors are presented.The expression levels of ASH1L in metastatic tumors (SU2C) containing ASH1L shallow deletion, diploid, gain, and amplification were compared using the unpaired Student's t-test. Overall survival data on 444 patients with metastatic PCa (SU2C) were downloaded from cbioportal. The survival data of patients with amplified or diploid ASH1L was compared using the log-rank (Mantel-Cox) test. The P value and hazard ratio (log-rank) were calculated using GraphPad Prism version 9.2.0.<br>To determine the association between ASH1L high expression and metastatic signatures in human PCa, we downloaded the RNA-seq RSEM dataset from the PCa TCGA project (493 cases). Samples were ranked by ASH1L expression and classified into three groups: ASH1L-high (150 cases), ASH1L-medium (193 cases), and ASH1L-low (150 cases). The CHANDRAN METASTASIS gene set was analyzed in ASH1L-high versus

ASH1L-low samples using GSEA 4.1.0. HIF-1α target gene enrichment analysis was performed using GSEA 4.1.0. Pearson correlation between ASH1L and HIF1A mRNA levels in PCa (TCGA dataset) was obtained from cbioportal.
The published scRNA-seq datasets of primary PCa (n=13 patients; GSE141445) and metastatic PCa (n=13 patients; GSE210358) were analyzed using Bioturing (https://academic.bioturing.com/login).
The bulk RNA-seq and CNA datasets of 208 metastatic PCa samples were downloaded from cbioportal and uploaded to CIBERSORTx (https://cibersortx.stanford.edu) to generate the proportions of immune cells in metastatic PCa tumors.
Living Image software (Caliper Life Sciences) were used for analyzing Bioluminescence signals were analyzed.
For RNA-seq, the reads were aligned to the human genome (hg38) using TopHat (version 2.0.10)(PMID: 23618408). The statistical assessment of differential expression between conditions was performed using the R/Bioconductor package DESeq (version 1.18.0)(PMID: 20979621). IPA was used to analyze pathway.
For CUT&RUN, 3' ends adapter sequences were removed using Trim Galore! (Version 0.6.5) and Cutadapt (Version 2.8). The reads were then mapped to the spike-in genome (sacCer3) initially, and the remaining reads were subsequently mapped to the human genome (hg38) using Bowtie (version 1.1.2) with the following parameters: "--allow-contain --maxins 2000 -v 2 -m 1 --best --strata". Differential analysis was performed by the R/Bioconductor package edgeR.
IGV was used to visualize the peaks and alignments for RNA-seq and CUT&RUN.
scRNA-seq data from bone metastasis was processed by CellRanger 6.1.2 (10X Genomics) and then analyzed using Bioturing (https://academic.bioturing.com/login)
Qupath software version 0.5.1-x64 was used for bone volume fraction and osteoblasts quantification.
Detailed information can be found in Method section.

For manuscripts utilizing custom algorithms or software that are central to the research but not yet described in published literature, software must be made available to editors and reviewers. We strongly encourage code deposition in a community repository (e.g. GitHub). See the Nature Portfolio guidelines for submitting code & software for further information.

Data

Policy information about availability of data

All manuscripts must include a data availability statement. This statement should provide the following information, where applicable:

- Accession codes, unique identifiers, or web links for publicly available datasets
- A description of any restrictions on data availability
- For clinical datasets or third party data, please ensure that the statement adheres to our policy

The raw data and processed data for scRNA-seq (GSE269895, https://www.ncbi.nlm.nih.gov/geo/query/acc.cgi?acc=GSE269895), bulk RNA-seq (GSE269830, https://www.ncbi.nlm.nih.gov/geo/query/acc.cgi?acc=GSE269830), and CUT&RUN-seq (GSE269829, https://www.ncbi.nlm.nih.gov/geo/query/acc.cgi?acc=GSE269829) generated in this study have been deposited in the GEO repository and released to the public. Public scRNA-seq data used in this study are available from GEO under the following accession code: GSE141445 (scRNA-seq data of 13 primary PCa patients) and GSE210358 (scRNA-seq data of 13 metastatic PCa patients). The bulk RNA-seq dataset was downloaded from cBioPortal (https://www.cbioportal.org/). All data are available in the main text or the Supplementary Information. Source data are provided in this paper.

Human research participants

Policy information about studies involving human research participants and Sex and Gender in Research.

Reporting on sex and gender	N/A
Population characteristics	N/A
Recruitment	N/A
Ethics oversight	N/A

Note that full information on the approval of the study protocol must also be provided in the manuscript.

Field-specific reporting

Please select the one below that is the best fit for your research. If you are not sure, read the appropriate sections before making your selection.

☒ Life sciences ☐ Behavioural & social sciences ☐ Ecological, evolutionary & environmental sciences

For a reference copy of the document with all sections, see nature.com/documents/nr-reporting-summary-flat.pdf

Life sciences study design

All studies must disclose on these points even when the disclosure is negative.

Sample size: No sample size calculation was performed. Sample size was determined based on previous published reports and experimental experience for in vivo and in vitro studies.Sample sizes were chosen based on empirical values that were sufficient to detect meaningful biological

	differences with good reproducibility.
Data exclusions	For the analysis of metastasis, the mice without whole body signals at Day 0 after intra-cardiac injection were excluded.<br>For analysis of IVIS imagining, the signals with unreasonable reduction at some time point were excluded.
Replication	For animal experiments, multiple independent repeats (individual mice) were included. For other experiments, biological repeats were used and each experiment was repeated at least three times with similar results. All attempts at replication were successful.
Randomization	Mice were randomized before injection with different cell lines.
Blinding	The investigators were not blinded to allocation during the experimental procedures and the assessment of the outcomes. The investigators were not blinded for the allocation of groups during experiments or during in vitro or in vivo experiments as the same investigator was responsible for setting-up and performing the experiment. Fully blinded animal experiments were not possible due to personnel availability to accommodate such situations.

Reporting for specific materials, systems and methods

We require information from authors about some types of materials, experimental systems and methods used in many studies. Here, indicate whether each material, system or method listed is relevant to your study. If you are not sure if a list item applies to your research, read the appropriate section before selecting a response.

Materials & experimental systems

n/a	Involved in the study
☐	☒ Antibodies
☐	☒ Eukaryotic cell lines
☒	☐ Palaeontology and archaeology
☐	☒ Animals and other organisms
☒	☐ Clinical data
☒	☐ Dual use research of concern

Methods

n/a	Involved in the study
☒	☐ ChIP-seq
☒	☐ Flow cytometry
☒	☐ MRI-based neuroimaging

Antibodies

Antibodies used	All antibodies used are commercially available and validated by the manufacturers.<br>Antibodies used for IHC, IF and Multiplex IHC are as below:<br>anti-ASH1L (1:50, Bethyl, A301-748A, RRID: AB_1210879);<br>anti-HA (1:800, Cell Signaling Technology, 3724S, RRID: AB_1549585);<br>anti-MMR/CD206 (1:100, R&D Systems, AF2535, RRID: AB_2063012);<br>anti-F4-80 (1:100, Cell Signaling Technology, 70076, RRID: AB_2799771);<br>anti-Ly-6C (1:100, Biolegend, 128001, RRID:AB_1134214).<br>anti-Ly-6C (1:100, Abcam, ab314120).<br><br>Antibodies used for Western blot and IP are as below:<br>anti-β-actin (1:5000, Sigma-Aldrich, A5441-.2ML, RRID: AB_476744);<br>anti-H3K4me3 (1:2000, Cell Signaling Technology, 9751S, RRID: AB_2616028);<br>anti-H3K36me3 (1:2000, Cell Signaling Technology, 4909S, RRID: AB_1950412);<br>anti-H3K36me2 (1:2000, Abcam, ab9049, RRID: AB_1280939);<br>anti-histone H3 (Cell Signaling Technology,4499S, RRID: AB_1280939);<br>anti-HA (1:1000, Cell Signaling Technology, 3724S, RRID: AB_1549585);<br>anti-HIF1A (1:1000, Cell Signaling Technology, 36169, RRID: AB_2799095);<br>anti-ASH1L (2ug for IP, 1:1000 for Western blot, Bethyl, A301-749A, RRID: AB_1210880);<br>anti-p-AKT (1:1000, Cell Signaling Technology, 4060S, RRID:AB_2315049);<br>anti-AKT (1:1000, Cell Signaling Technology, 4691S, PRID: AB_915783);<br>anti-p-ERK (1:1000, Cell Signaling Technology, 9101S, PRID: AB_331646);<br>anti-ERK(1:1000, Cell Signaling Technology, 9102S, PRID: AB_330744);<br>anti-rabbit IgG (HRP) (1:5000, Cell Signaling Technology, 7074V, RRID: AB_2099233);<br>anti-mouse IgG (HRP) (1:5000, Cell Signaling Technology, 7076V, RRID: AB_330924).<br><br>Antibodies used for CUT&RUN are as below:<br>Rabbit (DA1E) mAb IgG XP® Isotype Control (5ul per reaction, Cell Signaling Technology,66362, RRID: AB_2924329);<br>anti-H3K4me3 (2ul per reaction, Cell Signaling Technology, 9751S, RRID: AB_2616028);<br>anti-H3K36me3 (2ul per reaction, Abcam, ab9050,RRID: AB_306966).
Validation	ASH1L antibody (Bethyl, A301-749A), the validation was provided by the manufacturer's website and also performed by using PC3M and DX1 mouse cell lines with CRISPER/CAS9 mediated ASH1L depletion vs control by Western blot.

anti-H3K4me3 (Cell Signaling Technology, 9751S, RRID: AB_2616028) have been validated in our previous publications: PMID: 2816653. For anti-rabbit IgG (HRP) (Cell Signaling Technology, 7074V, RRID: AB_2099233); anti-mouse IgG (HRP) (Cell Signaling Technology, 7076V, RRID: AB_330924) and anti-β-actin (Sigma-Aldrich, A5441-.2ML, RRID: AB_476744) have been validated in our previous publications: PMID: PMID: 35771632 and PMID: 37163614.

The validation of other antibodies was provided by the manufacturer's website:
anti-ASH1L (Bethyl, A301-748A, RRID: AB_1210879): https://www.thermofisher.com/antibody/product/ASH1-Antibody-Polyclonal/A301-748A
anti-HA (Cell Signaling Technology, 3724S, RRID: AB_1549585): https://www.cellsignal.com/products/primary-antibodies/ha-tag-c29f4-rabbit-mab/3724?site-search-type=Products&N=4294956287&Ntt=3724s&fromPage=plp&_requestid=181116
anti-H3K36me3 (Cell Signaling Technology, 4909S, RRID: AB_1950412): https://www.cellsignal.com/products/primary-antibodies/tri-methyl-histone-h3-lys36-d5a7-xp-rabbit-mab/4909?site-search-type=Products&N=4294956287&Ntt=4909s&fromPage=plp&_requestid=343747
anti-H3K36me3 (Abcam, ab9050, RRID: AB_306966): https://www.abcam.com/products/primary-antibodies/histone-h3-tri-methyl-k36-antibody-chip-grade-ab9050.html
anti-H3K36me2 (Abcam, ab9049, RRID: AB_1280939): https://www.abcam.com/products/primary-antibodies/histone-h3-di-methyl-k36-antibody-chip-grade-ab9049.html
anti-histone H3 (Cell Signaling Technology,4499S, RRID: AB_1280939): https://www.cellsignal.com/products/primary-antibodies/histone-h3-d1h2-xp-rabbit-mab/4499?site-search-type=Products&N=4294956287&Ntt=4499s&fromPage=plp&_requestid=343819
HIF1A (Cell Signaling Technology, 36169, RRID: AB_2799095): https://www.cellsignal.com/products/primary-antibodies/hif-1a-d1s7w-xp-rabbit-mab/36169
anti-MMR/CD206 (R&D Systems, AF2535, RRID: AB_2063012): https://www.rndsystems.com/products/mouse-mmr-cd206-antibody_af2535
anti-F4-80 (Cell Signaling Technology, 70076, RRID: AB_2799771): https://www.cellsignal.com/products/primary-antibodies/f4-80-d2s9r-xp-174-rabbit-mab/70076
anti-Ly-6C (Biolegend, 128001, RRID:AB_1134214): https://www.biolegend.com/en-gb/products/purified-anti-mouse-ly-6c-antibody-4894
Rabbit (DA1E) mAb IgG XP® Isotype Control (Cell Signaling Technology,66362, RRID: AB_2924329): https://www.cellsignal.com/products/primary-antibodies/rabbit-da1e-mab-igg-xp-isotype-control-cut-amp-run/66362

Eukaryotic cell lines

Policy information about cell lines and Sex and Gender in Research

Cell line source(s)	293T (CRL-3216), DU145 (HTB-81), PC-3 (CRL-1435) , LNCaP (CRL-1740) and THP-1 (TIB-202) cell lines were obtained from the American Type Culture Collection (ATCC). B16F1, B16F10, MDA-MB-231, MDA-231-LM2, and MDA-231-BoM-1833 melanoma and breast cancer cell lines were gifts from Dr. Li Ma's laboratory (MD Anderson Cancer Center, Houston, TX). PC-3M cell lines were gifts from Dr. Ronald DePinho's laboratory (MD Anderson Cancer Center, Houston, TX). Murine primary and LN metastatic cells were generated from our male GEMM model and validated by western blot. DX1 mouse prostate cancer cells were generated from our GEMM model and validated by western blot.<br>Human primary monocytes were isolated from Peripheral blood of healthy donors (males and females)(MD Anderson Blood Donor Center)
Authentication	Short tandem repeat (STR) profiling was done by ATCC and MD Anderson's Cytogenetics and Cell Authentication Core.
Mycoplasma contamination	All of the cell lines were tested to be negative for mycoplasma using MycoAlert PLUS detection kit (Lonza, LT07-710)
Commonly misidentified lines (See ICLAC register)	No commonly misidentified lines were used

Animals and other research organisms

Policy information about studies involving animals; ARRIVE guidelines recommended for reporting animal research, and Sex and Gender in Research

Laboratory animals	Male nude mice and male C57BL/6 mice at 4 weeks of age were purchased from from Taconic Bioscience. Mice were used for experimental studies at 6-8 week of age. The Pb-Cre; PtenL/L; Trp53L/L; Smad4L/L; mTmG (PbPPS) mouse strain was a gift from Dr. Ronald DePinho's research group at MD Anderson (Houston, TX). Mice were interbred, regularly genotyped, and maintained at the MD Anderson animal facility. All nude mice used in this study are the strain of CrTac:NCr-Foxn1nu (Sp/Sp) purchased from Taconic Bioscences (Cat # NCRNU-M). All C57BL/6 mice used in this study were purchased from Taconic. Due to the nature of the PCa study, only male mice were used in this study. All mouse experimental procedures followed the Institutional Animal Care and Use Committee (IACUC) protocol (#00001955). The mice were housed under a 12-hour light/dark cycle, at 68-78°F with 30–70% humidity. MD Anderson IACUC's guidelines for the proper and humane use of animals in biomedical research were followed.
Wild animals	The study did not involve wild animals.
Reporting on sex	Due to the nature of the PCa study, only male mice were used in this study.
Field-collected samples	To establish primary prostate cancer xenograft model, tumors on flank(s) of mice were collected. For metastatic models, bone or brain samples were collected. For GEMM model, prostate and metastatic tumors was collected. All mice were interbred and maintained at MD Anderson Cancer Center, monitored for signs of ill health every day, and sacrificed and necropsied when

	moribund.
Ethics oversight	All manipulations were performed under the review and approval of MD Anderson Cancer Center's Institutional Animal Care and Use Committee (Protocol #00001955).

Note that full information on the approval of the study protocol must also be provided in the manuscript.
